# The therapeutic potential of traditional Chinese medicine in depression: focused on the modulation of neuroplasticity

**DOI:** 10.3389/fphar.2024.1426769

**Published:** 2024-08-26

**Authors:** Shimeng Lv, Ni Yang, Yitong Lu, Guangheng Zhang, Xia Zhong, Yaru Cui, Yufei Huang, Jing Teng, Yanyan Sai

**Affiliations:** ^1^ Department of First Clinical Medical College, Shandong University of Traditional Chinese Medicine, Jinan, China; ^2^ Institute of Child and Adolescent Health, School of Public Health, Peking University, Beijing, China; ^3^ Innovative Institute of Chinese Medicine and Pharmacy, Shandong University of Traditional Chinese Medicine, Jinan, China; ^4^ Ruijin Hospital Affiliated to Shanghai Jiaotong University School of Medicine, Shanghai, China; ^5^ University Town Hospital, Afiliated Hospital of Shandong University of Traditional Chinese Medicine, Jinan, China

**Keywords:** depression, major depressive disorder, antidepressant, traditional Chinese medicine, neuroplasticity

## Abstract

Depression, a mood disorder characterized by a persistent low mood and lack of enjoyment, is considered the leading cause of non-fatal health losses worldwide. Neuroplasticity refers to the brain’s ability to adapt to external or internal stimuli, resulting in functional and structural changes. This process plays a crucial role in the development of depression. Traditional Chinese Medicine (TCM) shows significant potential as a complementary and alternative therapy for neurological diseases, including depression. However, there has been no systematic summary of the role of neuroplasticity in the pathological development of depression and TCM Interventions currently. This review systematically summarized recent literature on changes in neuroplasticity in depression and analyzed the regulatory mechanisms of active metabolites in TCM and TCM formulas on neuroplasticity in antidepressant treatment. Additionally, this review discussed the limitations of current research and the application prospects of TCM in regulating neuroplasticity in antidepressant research.

## 1 Introduction

Depression is a mood illness marked by enduring feelings of sadness and lack of enjoyment. The global average incidence rate is about 4.4%. By 2030, depression is expected to become the leading cause of disease burden worldwide, being the primary contributor to non-fatal health loss globally (Rehm and Shield, 2019; Bayes et al., 2020). Selective serotonin reuptake inhibitors (SSRIs) and other Western medicine therapies are the mainstays of treatment; however, most medications have delayed effects, high rates of non-responsiveness, and significant side effects such as headaches, nausea, weight gain, and chronic dysfunction (Wang et al., 2019; Qu et al., 2021; Wei et al., 2022).

Therefore, developing more effective and safer antidepressant drugs has become an urgent problem to be solved. Traditional Chinese Medicine (TCM) has a long history of understanding and treating depression. TCM is known for its multi metabolite, multi target, multi link, and multi pathway characteristics, which can act on multiple aspects of the disease and have high efficacy and low toxicity. This highlights the advantages and good prospects of TCM in treating depression. Importantly, compared to Western medicine, they have the advantages of easy use, good therapeutic effects, minimal dosage, and fewer side effects. Due to the shortcomings of existing antidepressants and the urgent market demand, research on the antidepressant mechanism of TCM has attracted much attention (Zhuang et al., 2023).

Neuroplasticity refers to the brain’s ability to respond to external or internal stimuli from the environment or organs, resulting in functional and structural changes (Vints et al., 2022). Neuroplasticity is closely related to depression (Tartt et al., 2022), and is a significant focus for the development of future antidepressant drugs (Duman et al., 2016). However, there remains a notable lack of a systematic overview regarding the role of neuroplasticity in the pathological development of depression and the intervention of TCM.

Based on the above findings, this review systematically summarized the changes in neuroplasticity observed in clinical and preclinical studies of depression by searching relevant literature from recent years. Furthermore, it explored into the pharmacological mechanisms through which TCM modulated neuroplasticity to treat depression, providing scientific basis for subsequent basic research and clinical applications.

## 2 Review methodology

To investigate how TCM exerted antidepressant effects by regulating neuroplasticity, we conducted a comprehensive search of articles in PubMed, Embase, Web of Science, and ScienceDirect databases. The search keywords included “Traditional Chinese Medicine,” “Chinese herbal medicine,” “herb,” “Traditional Chinese Medicine formulas,” “Traditional Chinese Medicine metabolites,” “depression,” “major depressive disorder,” “syntactic plasticity,” and “neuroplasticity.” The retrieved articles were reviewed by two independent reviewers based on their titles, abstracts, and full texts, adhering to specific inclusion and exclusion criteria. The inclusion criteria were: 1) Original articles written in English; 2) Articles that examined the relevant mechanisms of TCM in regulating neuroplasticity for the treatment of depression. Exclusion criteria were as follows: 1) Articles written in any language other than English; 2) Gray literature; 3) Editorials; 4) Review articles; 5) Duplicate publications.

## 3 Overview of neuroplasticity

### 3.1 Definition of neuroplasticity

Neuroplasticity is a crucial concept in life sciences, describing how the brain changes and adapts to environmental changes by continually forming new neural connections (Price and Duman, 2020). It represents the adaptability of the nervous system, enabling it to adjust to learning, memory, environmental changes, and rehabilitation following brain injury. The main mechanisms include the regulation of synaptic strength, structural remodeling, and the regulation of intrinsic neuronal properties. These processes are dynamic, involving changes in the number of brain nuclei and structures, various functions, and numerous interactions (Xing and Bai, 2020; Dzyubenko and Hermann, 2023). Neuroplasticity is essential for understanding brain development, learning, and the regulation of homeostasis in the central nervous system (CNS).

### 3.2 Classification of neuroplasticity

**FIGURE 1:**
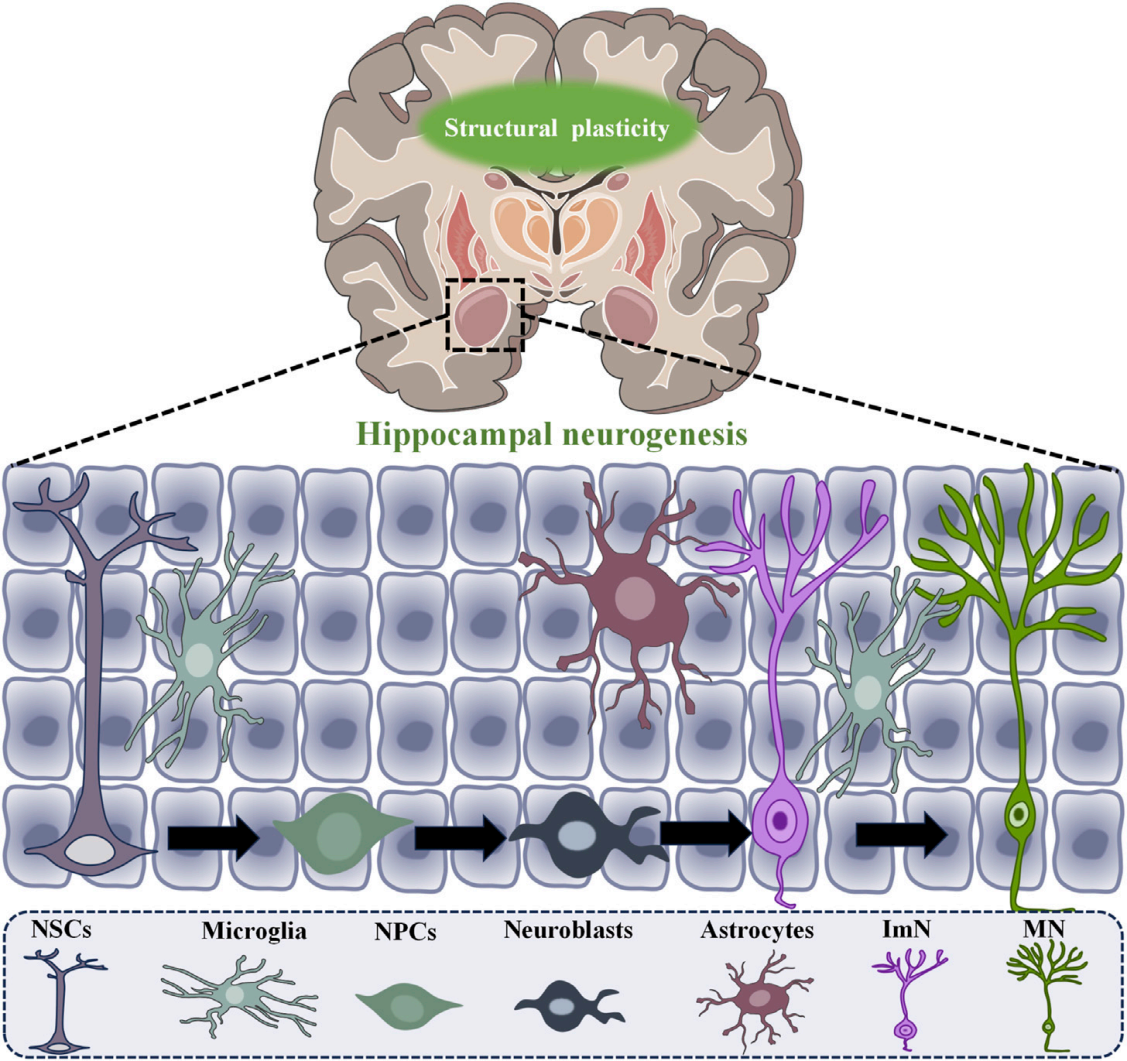
Schematic diagram of neurogenesis in structural plasticity.

Neuroplasticity includes two primary types: structural plasticity and functional plasticity. Structural plasticity refers to changes in mechanisms that promote neurogenesis, the formation of dendritic spines, and the growth and repair of axons. It includes changes in the number and connectivity of synapses, the density of dendritic spines, and modifications in neural processes like axons and dendrites, as well as variations in the number of neuronal cells (De Paola et al., 2006; Knott et al., 2006). On the other hand, functional plasticity involves synaptic changes between neurons without modifying their physical structure, such as long-term potentiation (LTP) and long-term depression (LTD) effects (Castillo, 2012; Marsden, 2013; Diering and Huganir, 2018). LTP and LTD are crucial mechanisms that affect cognitive and emotional functions in depression patients. Intense and sustained stimulation leads to an increase in neuronal discharge, which in turn enhances the strength of synapses. This process facilitates learning and memory, thereby promoting LTP. In contrast, LTD is characterized by a decrease in the efficacy and connectivity of neuronal synapses (Figure 1 showed a schematic diagram of neurogenesis).

**FIGURE 2:**
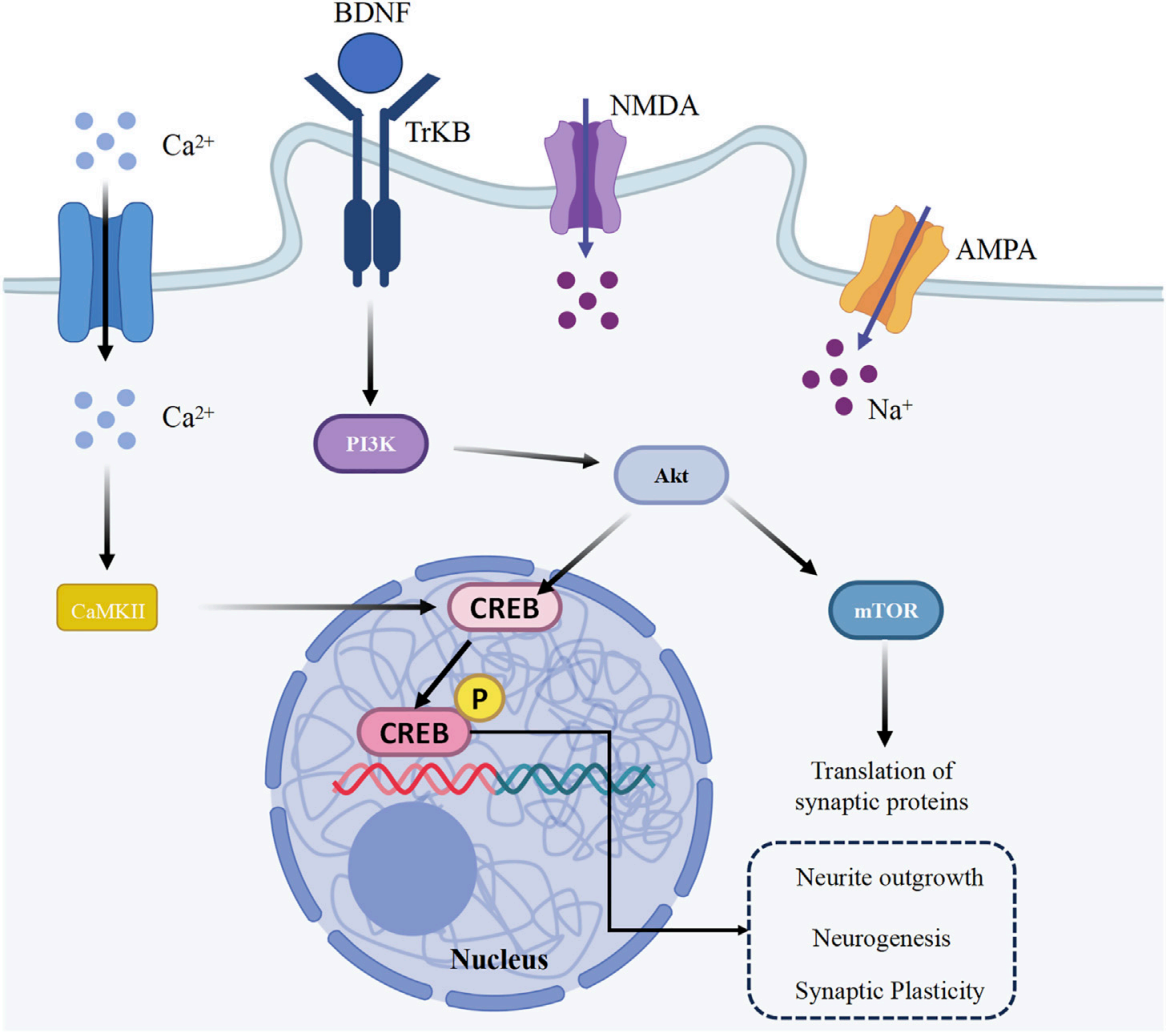
Neuroplasticity regulatory mechanisms.

Neuroplasticity is regulated by several key mechanisms, one of which is the brain-derived neurotrophic factor (BDNF)/tyrosine kinase receptor B (TrkB) signaling pathway. The synthesis of BDNF is triggered by the activation of cyclic adenosine monophosphate (cAMP) responsive element binding protein (CREB). CREB is pivotal in facilitating LTP and synaptic plasticity. When BDNF binds to TrkB receptors, it triggers various signaling cascades, such as the mitogen-activated protein kinase/extracellular signal-regulated kinase (MAPK/ERK), phosphoinositide 3-kinase (PI3K), and mammalian target of rapamycin (mTOR) pathways, which are responsible for spine enlargement and increased glutamate sensitivity (Figure 2 showed the regulatory mechanism) (Bourtchuladze et al., 1994; Tanaka et al., 2008; Tejeda and Díaz-Guerra, 2017).

## 4 Neuroplasticity and depression

### 4.1 Changes in neuroplasticity in depression

#### 4.1.1 Clinical studies

Meta-analysis is a prominent method for evaluating the effectiveness of public health interventions (Tanner-Smith and Grant, 2018). In the pathophysiology of depression, impaired neuroplasticity plays a crucial role, as indicated by a meta-analysis conducted on the cerebrospinal fluid of individuals with unipolar depression (Mousten et al., 2022). Studies have shown that the increase in motor evoked potential amplitude, induced by paired associative stimulation, weakens during severe depressive episodes and normalizes during remission. It suggests the presence of LTP deficits in individuals with depression (Player et al., 2013; Kuhn et al., 2016). Furthermore, compared to healthy subjects, patients with depression, particularly those with refractory depression, exhibit impaired neuroplasticity in the dorsolateral prefrontal cortex. Female patients with depression also demonstrate persistent LTD-like plasticity deficits (Noda et al., 2018; Yu et al., 2020; Kaneko et al., 2024). Abnormal changes in neuroplasticity-related proteins have been observed in depression patients (Hidese et al., 2020). The ratio of BDNF to leptin levels has been associated with treatment responses in depression and may also be related to the neuroplasticity of depression, as evidenced by a 12-week follow-up study (An et al., 2019).

#### 4.1.2 Preclinical studies

##### 4.1.2.1 Depression model induced by stress

Stress is recognized as a normal physiological and psychological response to both positive and negative situations. Chronic stress, in particular, plays a key role in the development of mental illnesses such as depression (Ray et al., 2017; Beurel et al., 2020; Monroe and Harkness, 2022). Prolonged exposure to chronic stress exacerbates the phagocytosis of synaptic elements and results in defects in neuroplasticity (Kokkosis et al., 2024). Synaptic pruning, as a developmental process, is closely related to synaptic plasticity. In models of depression induced by chronic unpredictable mild stress (CUMS), excessive activation of microglia leads to exaggerated synaptic pruning (Zhang et al., 2022a), accompanied by impairments in synaptic plasticity (Li et al., 2021a; Yan et al., 2021). Early-life stress increases susceptibility to depression in adolescent mice by regulating the miR-34c-5p/synaptotagmin-1 (SYT1) axis and disrupting hippocampal neuroplasticity (Yu et al., 2024). In a combined model, adult female rats subjected to maternal-infant separation (MS) and CUMS exhibited more severe depressive and anxiety-like behaviors, potentially linked to compromised synaptic plasticity (Huang et al., 2021). In a depression model where insomnia was induced by CUMS combined with sleep deprivation, dendritic spines in the hippocampal dentate gyrus (DG) region were damaged, neural networks were disrupted, and neuroplasticity was inhibited (Li et al., 2022). Studies have also demonstrated that the absence of bombesin receptor-activated protein homologous protein affects hippocampal synaptic plasticity and exacerbates CUMS-mediated behavioral changes (Yao et al., 2023). Mechanistic research has revealed that CUMS alters synaptic plasticity in the nucleus accumbens (NAc) by influencing Kv4.2 channels through glycogen synthase kinase 3β (GSK3β)-dependent mechanisms (Aceto et al., 2020). Additionally, CUMS can disrupt the synaptic plasticity of regenerating neurons in the hippocampus of ischemic rats via astrocytic glutamate transporter-1 (Yu et al., 2019).

##### 4.1.2.2 Depression model induced by social isolation

Social isolation can induce fatigue, behavioral changes, substance abuse, and various mental illnesses. These effects can be sustained and irreversible, impacting both humans and animals and increasing the risk of developing mental illness (Jaremka et al., 2014; Hueston et al., 2017). Neuroplasticity-related signals play a crucial role in the impact of induced isolation on sexual and neurological behavioral deficits (Liu et al., 2020). Animals subjected to chronic social isolation (CSIS) displayed depressive-like behavior (Perić et al., 2021), accompanied by proteomic findings showing dysregulated expression of synaptic plasticity-related proteins (Filipović et al., 2023). Moreover, animals raised in isolation exhibited immature dendritic spines that appear small and thin, with impaired neuroplasticity observed through LTP testing (Medendorp et al., 2018). However, treatment with fluoxetine has been shown to alleviate depressive-like behavior induced by CSIS and regulate neuroplasticity-related proteins (Filipović et al., 2022).

##### 4.1.2.3 Depression mode induced by corticosterone

The hypothalamic-pituitary-adrenal (HPA) axis is a vital metabolite of the neuroendocrine system. When active, the anterior pituitary gland releases adrenocorticotropin (ACTH) into the bloodstream. This signal is received by the paraventricular nucleus of the hypothalamus, which then produces corticotropin-releasing hormone. ACTH, in turn, stimulates the adrenal cortex to release cortisol (CORT) (Frankiensztajn et al., 2020). Excessive activation of the HPA axis correlates significantly with sustained elevation of CORT levels and depression. Elevated CORT levels observed in individuals with depression closely correlate with the severity of depressive symptoms and poor treatment outcomes (Karin et al., 2020). Chronic exposure to CORT reduces the structural plasticity of astrocytes in the hippocampus of mice, leading to hippocampal atrophy (Zhang et al., 2015). Mice treated with CORT exhibit depressive-like behavior accompanied by changes in synaptic plasticity (Crupi et al., 2013; Freitas et al., 2016). Mechanistic studies have shown that CORT reduces synaptic density and vesicle recycling by downregulating BNIP3 like (BNIP3L)/NIX, thereby inhibiting mitochondrial autophagy (Choi et al., 2021).

##### 4.1.2.4 Lipopolysaccharide (LPS) induced depression model

LPS can be found in the outer wall of Gram-negative bacterial cells, consisting of lipids and polysaccharides. Mouse models induced with LPS to mimic depression-like symptoms are commonly used to study the mechanisms of inflammation-related depression and the therapeutic effects of various drugs (Yin et al., 2023). Early reports indicated that LPS administration could induce LTP and depression in the hippocampal CA1 area (Jo et al., 2001). Recent studies have found that LPS mediates depressive-like behavior by promoting neuroinflammation in the basolateral amygdala (BLA), enhancing glutamatergic synaptic transmission, and increasing the intrinsic excitability of BLA projection neurons (Zheng et al., 2021). Wu et al. (2019), through a combination of proteomics and metabolomics, found that LPS intervention in mice disrupts glutamatergic transmission and Ephrin receptor signaling, potentially leading to impaired hypothalamic synaptic plasticity and depressive-like behavior.

### 4.2 Impact of antidepressant treatment on neuroplasticity

#### 4.2.1 Chemicals acting on the CNS

Fluoxetine, a widely used SSRI in clinical practice, exerts its antidepressant effects by enhancing synaptic plasticity (Qian et al., 2024). It alos modified mood behaviors and hippocampal neuroplasticity by disrupting the nNOS-CAPON interaction that links postsynaptic 5-HT1AR activation (Shi et al., 2022). Additionally, fluoxetine enhances hippocampal neuroplasticity by promoting axonal formation induced by growth-associated protein 43 (GAP-43) (Zavvari et al., 2020). Pre-treatment with fluoxetine has been shown to prevent stress-induced LTD and spatial memory deficits in the hippocampus of rats (Han et al., 2015). Citalopram, another SSRI, is composed of two enantiomers, R-citalopram and S-citalopram, which inhibit serotonin (5-HT) reuptake in the brain, thereby exerting an antidepressant effect (Yan et al., 2023a). When combined with *Punica granatum*, citalopram can alleviate damage to dendritic spines in the hippocampal DG region (Vega-Rivera et al., 2023).

Agomelatine, a synthetic analogue of melatonin, exerts its antidepressant effects by stimulating melatonin receptors (MT1 and MT2) and antagonizing 5-HT2C receptors (Maddukuri et al., 2021). Research indicates that agomelatine improves pathological behavior in stressed mice by modulating BDNF signaling, synaptic plasticity, and epigenetic remodeling (Martin et al., 2017). It also demonstrates beneficial effects in mitigating stress-induced brain damage, as it restores the activity of hippocampal neurons affected by stress and promotes adult hippocampal neurogenesis (Dagyte et al., 2010).

Ketamine, a non-competitive N-methyl-D-aspartate receptor (NMDAR) antagonist, specifically inhibits GluN2B-containing NMDARs on inhibitory GABAergic interneurons (Sato et al., 2022). Its antidepressant mechanism involves modulating neuroplasticity (Clarke et al., 2017), and low concentrations of ketamine (20 μM) can induce postsynaptic enhancement in the hippocampal CA1 (Kim and Monteggia, 2020). Its antidepressant effects are mediated by increased neuroplasticity, including synaptic actions (Kopelman et al., 2023), and it can “reset the system” by participating in synaptic plasticity processes to reverse stress-induced loss of key neural circuit connections (Aleksandrova et al., 2020). In animal models with chronic pain and depression, TIAM1-mediated synaptic plasticity is a crucial factor in the antidepressant effect of ketamine (Ru et al., 2022). Ketamine may also exert rapid antidepressant effects by enhancing neuroplasticity, triggering autophagy, and preventing ferroptosis in the nucleus (Zhang et al., 2022b). Studies have found that ketamine-induced hippocampal synaptic plasticity during antidepressant treatment depends on 4E binding proteins (Aguilar-Valles et al., 2021).

#### 4.2.2 Other types of chemicals

Metformin is the first-line treatment for type 2 diabetes, primarily acting by reducing liver gluconeogenesis and enhancing glucose metabolism. It also exhibits pleiotropic effects (LaMoia and Shulman, 2021). Beyond its antidiabetic role, metformin has been investigated for its potential in treating depression. Studies indicate that compared to other oral hypoglycemic drugs, metformin is associated with a lower risk of depression and demonstrates pleiotropic effects in depression management (Yu et al., 2022). Additionally, metformin can upregulate the expression of plasticity markers such as synapsin, sirtuin-1, AMP-activated protein kinase, and BDNF (Muñoz-Arenas et al., 2020). When combined with fluoxetine, metformin enhances the survival of NeuN-positive cells in the hippocampus and increases the number of BDNF-positive cells stimulated by fluoxetine, thereby enhancing its impact on neuroplasticity (Mendonça et al., 2022). Furthermore, metformin has been shown to mitigate synaptic plasticity damage induced by LPS in rats (Zhou et al., 2021) and improve the expression of synaptic plasticity markers [anti-microtubule-associated protein 2, synaptophysin (SYP), postsynaptic density protein 95], thereby alleviating depressive-like behavior in mice with allergic rhinitis (AR) (Lv et al., 2023). Hydrogen sulfide (H_2_S) is recognized as the third endogenous gas transmitter and can be produced in mammals through four enzyme pathways (Wu et al., 2018). H_2_S has been found to improve hippocampal synaptic plasticity in a Warburg-dependent manner, alleviating depression related to Parkinson’s disease (PD) (Liu et al., 2024) (Table 1).

**TABLE 1:** Regulation of antidepressant chemicals on neuroplasticity.

Chemical	Molecular formula	CAS NO.	Main mechanism of action	Reference
Fluoxetine	C_17_H_18_F_3_NO	5410-89-3	Enhanced synaptic plasticity	Qian et al. (2024)
			Modified mood behaviors and hippocampal neuroplasticity by disrupting the nNOS-CAPON interaction that links postsynaptic 5-HT1AR activation	Shi et al. (2022)
			Enhanced hippocampal neuroplasticity by promoting axonal formation induced by GAP-43	Zavvari et al. (2020)
			Preventing LTD and spatial memory deficits caused by stress	Han et al. (2015)
Citalopram	C_20_H_21_FN_2_O	59729-33-8	Combined use with *Punica granatum* can alleviate damage to dendritic spines in the DG region of the hippocampus	Vega-Rivera et al. (2023)
Agomelatine	C_15_H_17_NO_2_	138112-76-2	Modulating BDNF signaling, synaptic plasticity, and epigenetic remodeling	Martin et al. (2017)
			Restored the activity of hippocampal neurons affected by stress and promotes adult hippocampal neurogenesis	Dagyte et al. (2010)
Ketamine	C_13_H_16_ClNO	6740-88-1	Modulating neuroplasticity	Clarke et al. (2017)
			Inducing postsynaptic enhancement in the hippocampal CA1 region	Kim and Monteggia (2020)
			Increased neural plasticity, including synaptic interactions	Kopelman et al. (2023)
			Prevent the loss of critical neural circuit connections caused by stress	Aleksandrova et al. (2020)
Metformin	C_4_H_11_N_5_	657-24-9	Upregulation of the expression of plasticity markers such as synaptic proteins, deacetylase-1, AMP activated protein kinase, and BDNF	Muñoz-Arenas et al. (2020)
			Improved the survival rate of hippocampal NeuN positive cells and increase the number of BDNF positive cells stimulated by fluoxetine, thereby enhancing its effect on neural plasticity	Mendonça et al. (2022)
			Improving synaptic plasticity damage	Zhou et al. (2021)
			Improving the expression of synaptic plasticity markers	Lv et al. (2023)
H_2_S	H_2_S	7783-06-4	Improving synaptic plasticity in hippocampus	Liu et al. (2024)

In summary, neuroplasticity undergoes alterations in patients with depression and is also impaired in stress-induced, social isolation-induced, CORT-induced, and LPS-induced depression models. Substances like fluoxetine, ketamine, and metformin can mitigate depressive symptoms by modulating neuroplasticity, suggesting that targeted manipulation of neuroplasticity offers potential for treating depression. However, fluoxetine carries specific adverse effects in therapeutic contexts, including the potential for hallucinations, hepatotoxicity, neurotoxicity, and addiction, which may restrict its clinical application.

## 5 Pharmacological mechanisms of TCM

At present, the treatment of depression is a major issue in the medical field, and neuroplasticity is closely related to depression. Regulation based on neuroplasticity is one of the potential important measures for the treatment of depression. However, the current Western medicine for treating depression is mainly developed based on the “monoamine neurotransmitter hypothesis” of depression, but drug dependence and withdrawal reactions are common. Therefore, the development of new antidepressant drugs has become a hot topic at present. TCM has unique advantages in preventing and treating depression, including its overall concept, syndrome differentiation, and treatment methods, as well as the specific characteristics of its multiple components and targets, which are beneficial to the overall internal environment while treating depression (Zhuang et al., 2023). Numerous studies have shown that the active metabolites and herbal formulas in TCM are involved in regulating neuroplasticity during the process of antidepressant treatment.

### 5.1 Active metabolites of TCM

#### 5.1.1 Flavonoids

Engeletin, a flavonoid metabolite initially extracted from the leaves of *Astragalus mongholicus* Bunge (Huang et al., 2011), is a potent natural metabolite with antioxidant and anti-inflammatory properties (Fang et al., 2023). Recent research has shown that Engeletin exerts antidepressant effects by activating the BDNF/TrkB/mTORC1 signaling pathway and enhancing synaptic plasticity in the prefrontal cortex (Xu et al., 2023). Baicalein, an important flavonoid found in the roots *Scutellaria baicalensis* Georgi, is frequently used in Chinese medicine and herbal tea preparations to promote wellbeing (Chandrashekar and Pandi, 2022). In preclinical studies of antidepressant effects, baicalein has been found to activate the BDNF/TrkB/CREB signaling pathway and protect against synaptic plasticity damage in mice with depression related to PD (Zhao et al., 2021). It also increases the ratio of mature BDNF (mBDNF) to proBDNF, regulates neuronal survival and synaptic plasticity, and suppresses neuroinflammation, effectively alleviating LPS-induced depressive symptoms in mice (Liu et al., 2022). Baicalin, extracted from *S. baicalensis* Georgi, has significant biological activity, including anti-inflammatory properties (Guo et al., 2019). Its antidepressant effect involves regulating the expression of synaptophysin (SYP), PSD95, BDNF, and TrkB, activating the Rac1-cofilin pathway, and enhancing synaptic plasticity (Lu et al., 2019).

Quercitrin, a naturally occurring flavonoid found in various fruits and vegetables, is commonly used as a dietary metabolite and supplement (Chen et al., 2022a). In mice with LPS-induced depression, quercitrin intervention could improve hippocampal damage, restore the abnormal expression of the pCREB/BDNF/PSD95/Synapsin1 pathway, regulates the PI3K/AKT/NF-κB signaling pathway, and enhances neuroplasticity (Sun et al., 2021). Luteolin, another natural flavonoid found in plants such as *Chrysanthemum indicum* L., *Capsicum annuum* L., and *Perilla frutescens* (L.) Britton has been studied for its pharmacological mechanism in treating late-onset depression, involving the regulation of neuroplasticity-related proteins (Li et al., 2021b; Liu et al., 2023; Rauf et al., 2024).

Soy isoflavones (SI), essential metabolites of *Glycine max* (L.) Merr., have different biological functions. SI can upregulate the expression of phosphorylated SYP (p-SYP) and PSD95 in the hippocampus of mice, inhibit neuroinflammation, regulate tryptophan metabolism, and reverse LPS-induced depressive behavior (Lu et al., 2022). Additionally, S-equol, a metabolite of dietary soy isoflavones, has demonstrated antidepressant effects by increasing synaptic plasticity proteins and inhibiting neuroinflammation (Lu et al., 2021). Silibinin, a polymorphic flavonoid extracted from milk thistle [*Silybum marianum* (L.) Gaertn.] (Ma et al., 2023), exerts its antidepressant effects by improving neuroplasticity and increasing neurotransmitter levels (Yan et al., 2015).

#### 5.1.2 Polyphenols

Polyphenols, metabolites widely distributed in a variety of plants, have garnered significant interest for their potential pharmacological actions, particularly their immune-stimulating and anticancer activities (Wang et al., 2022a). These metabolites have been found to enhance brain function by directly influencing cells and processes in the CNS (Grabska-Kobyłecka et al., 2023). Curcumin,a primary bioactive polyphenolic metabolite extracted from the rhizomes of *Curcuma longa* L., has been extensively studied for its therapeutic properties. In an ovariectomy-induced depression model, Curcumin was found to be a safe and effective regulator of 5-HT, similar to fluoxetine and neurotrophic E2, and was involved in regulating neuroplasticity (Abd-Rabo et al., 2019; Zia et al., 2021). Additionally, in the CUMS model, Curcumin was found to improve depressive-like behavior in animals by regulating the expression of synaptic plasticity proteins (Zhang et al., 2014).

#### 5.1.3 Alkaloids

The defining characteristic uniting the diverse class of chemicals known as alkaloids is the presence of a nitrogen atom in a heterocyclic ring (Ziegler and Facchini, 2008). Berberine, an isoquinoline alkaloid derived from the Chinese botanical drug *Coptis chinensis* Franch. and related *Berberis* species, possesses a broad variety of pharmacological effects (Song et al., 2020). Studies have shown that Berberine effectively treats depression by inhibiting neuroinflammation and improving gut microbiota (Zhu et al., 2017; Yang et al., 2023a). Berberine has multi-target and multi-pathway antidepressant characteristics (Gao et al., 2024). Recent research has emphasized the impact of Berberine on neuroplasticity in the context of depression. In mouse models of depression treated with Berberine, an increase in neuronal and synaptic plasticity has been observed. Berberine targets enzymes such as tryptophan 5-hydroxylase one and indoleamine 2,3-dioxygenase one involved in tryptophan metabolism, thereby improving depressive symptoms in CUMS stimulated mice (Ge et al., 2023). Additionally, Berberine’s antidepressant effect is accompanied by a reduction in neuroinflammatory responses through the inhibition of NLRP3 inflammasome activation, promoting plasticity and neurogenesis to alleviate neuronal damage (Qin et al., 2023).

#### 5.1.4 Saponins

Saponins, naturally occurring substances found in a wide range of plants, have garnered interest for their potential pharmacological properties (Zhang et al., 2023). Saikosaponin C, a metabolite purified from the traditional Chinese botanical drug *Bupleurum chinense* DC., has been studied for its effects on depression. Recent reports indicate that saikosaponin C reduces IL6 levels by inhibiting DNA methyltransferase one protein, leading to a decrease in IL6 expression. This metabolite promotes synaptic plasticity and alleviates depression-like behavior induced by chronic social defeat stress (Pan et al., 2019; Bai et al., 2023).

Ginsenoside Rb1, one of the main ginsenosides found in *Panax ginseng* C.A.Mey., is known for its neuroprotective properties (Ni et al., 2022). Research has shown that ginsenoside Rb1 can alleviate depressive symptoms induced by CUMS by modulating hippocampal synaptic plasticity through the miR-134-mediated BDNF signaling pathway (Wang et al., 2022b). Additionally, ginsenoside Rb1 regulates mitochondrial autophagy and the NF-κB pathway to inhibit astrocyte apoptosis, thereby reducing neuroinflammation and enhancing synaptic plasticity to maintain nervous system homeostasis (Li et al., 2023a). Ginsenoside Rg1, another key metabolite of *P. ginseng* C.A.Mey., has gained attention for its potential in preventing neurological diseases, especially dementia and depression (Yang et al., 2023b). It has been found to synergize with exercise in treating depression by reducing inflammation and improving neuroplasticity (Wang et al., 2023a).

#### 5.1.5 Terpenoids

The largest class of natural products is terpenoids, offering a plethora of potential therapeutic candidates (Huang et al., 2012). *Gardenia jasminoides* J. Ellis contains a type of iridoid glycoside called geniposide, which has various of biological benefits, including anti-neurodegenerative effects (Shen et al., 2020a). In a mouse model of *postpartum* depression, researchers observed excessive activation of the HPA axis and abnormal expression of proteins related to synaptic plasticity. Treatment with geniposide can alleviate these pathological phenomena and improve depressive-like behavior in mice (Ma et al., 2024). Xia et al. (2021) found that iridoids from *Gardeniae fructus* exerted antidepressant-like effects by stimulating AMPAR/mTOR signaling to enhance synaptic plasticity. For over a millennium, *Paeonia lactiflora* Pall. has been used in TCM to address ailments related to pain, inflammation, and the immune system (Zhang and Wei, 2020). *Paeonia lactiflora* Pall. produces a water-soluble monoterpene glycoside known as paeoniflorin (Cao et al., 2023), effectively reversing LTP damage induced by CUMS in the hippocampal CA1 region. Additionally, it can prevent CUMS-induced changes in dendritic spine density in the mouse hippocampus and downregulate BDNF and postsynaptic density protein 95 (PSD95) expression (Liu et al., 2019).

The pentacyclic triterpenoid chemical oleanolic acid (OA) is a naturally occurring substance extracted from various plants, including *Olea europaea* L. (Luo et al., 2024). Ursolic acid (UA) is another naturally occurring pentacyclic triterpenoid found in plants (Li et al., 2023b). Kong et al. (2023) conducted a study comparing the antidepressant effects of OA and UA, and found that in a depression model induced by CMS, OA was more effective than UA at reversing the depressive-like behavior induced by MS. In their mechanistic study, it was found that both OA and UA treatments reversed the decrease in synapsin expression levels caused by MS, but only OA upregulated the expression level of PSD-95 (Kong et al., 2023).

#### 5.1.6 Polysaccharides

Polysaccharides are carbohydrate polymers composed of at least ten monosaccharides linked by glycosidic linkages (Yi et al., 2020). They are found in plants, microbes, bacteria, fungi, and seaweed all contain polysaccharides, playing crucial roles in various physiological processes (Chen et al., 2017). Post-traumatic stress disorder (PTSD) is a type of depression syndrome, and Xie et al. (2024) found that polysaccharides from *Polygonatum cyrtonema* Hua can improve PTSD-induced behavioral abnormalities and synaptic damage in mice by reducing oxidative stress and neuroinflammation, and by acting on the Nrf2/HO-1 signaling pathway. Inulin, a non-digestible fructan-type carbohydrate, was originally isolated from the roots of *Inula helenium* L. (Illippangama et al., 2022). Studies suggest that inulin improves neurogenesis and synaptic plasticity by enhancing CREB/BDNF signaling, prevents CUMS-induced reduction in blood-brain barrier permeability, reduces neuroinflammation, preserves intestinal barrier integrity, and promotes the production of short-chain fatty acids (SCFAs) (Wang et al., 2023b). *Schisandra chinensis* (Turcz.) Baill., belonging to the Magnoliaceae family and has been widely used as a medicinal plant in China for centuries. Modern pharmacological research has revealed the anti-inflammatory and anti-aging properties of *S. chinensis* and its active metabolites (Bian et al., 2022). Notably, studies have shown that the polysaccharide-rich fraction from *S. chinensis* (Turcz.) Baill. exhibits antidepressant effects in olfactory bulbectomized mice by enhancing abnormal synaptic plasticity (upregulating PSD95 expression), suppressing excessive activity of the HPA axis, and regulating gut microbiota (Zhu et al., 2024).

#### 5.1.7 Botanical drugs extracts

Botanical drugs extracts are considered valuable for their comprehensive active properties, driven by complex biochemical interactions and synergistic effects among their natural metabolites (Pace and Martinelli, 2022). Saffron (*Crocus sativus* L.), a well-known natural product, has long been used to prevent and treat different disorders (Ghaffari and Roshanravan, 2019). In rats exposed to chronic mild stress (CMS), repeated administration of doses of 100 mg/kg and 200 mg/kg doses of Saffron Extract (Affron^®^) effectively normalized HPA axis dysregulation. Moreover, hypothalamic neuroplasticity showed a significant dose-dependent increase following treatment with Saffron Extract (Affron^®^) (Kim et al., 2023). Blueberry (*Vaccinium* spp.), a member of the *Vaccinium* genus, is recognized as one of the top five nutritious foods for humans and is often referred to as the “king of fruits.” This reputation has fueled considerable interest in the market for plant-based prebiotics (Duan et al., 2022). Blueberry Extract has demonstrated efficacy in alleviating depression-like behavior in LPS-induced mice. It also mitigates the increase in acetylcholinesterase (AChE) activity in the hippocampus induced by LPS and inhibits the mRNA expression of TNF-α, IL-1β, and IL-10 in the cerebral cortex following LPS administration, indicating a potential protective effect on neuroplasticity (Spohr et al., 2023).

#### 5.1.8 Other types of active metabolites

Honokiol is a versatile lignan metabolites naturally occurring in plants like *Magnolia grandiflora* L., known for its anti-inflammatory and neuroprotective effects (Rauf et al., 2021; Hu et al., 2023). Fan et al. (2022) found that the antidepressant mechanism of Honokiol involved the activation of the HIF-1α/VEGF signaling pathway and the upregulation of synaptic protein one and PSD 95 expression levels. Salidroside, an active metabolites found in *Rhodiola rosea* L. used in TCM, has various pharmacological effects (Xue et al., 2019). It enhances BDNF expression, improve synaptic plasticity, and inhibits pyroptosis mediated by the P2X7/NF-κB/NLRP3 signaling pathway, thereby providing a treatment for depression (Chai et al., 2022).

Crocin is a hydrophilic carotenoid produced in the blooms of *C. sativus* L., has been associated with promoting new nerve cell generation in the adult hippocampus and exerting antidepressant effects by activating the Wnt/β-catenin signaling pathway (Boozari and Hosseinzadeh, 2022; Tao et al., 2023a), Neurogenesis plays a key role in the physiological mechanism of structural neuroplasticity. Wu et al. (2020) also found that crocin rapidly and persistently induced antidepressant effects in mice subjected to Prenatal stress (PNS), acting through the GHSR-PI3K signaling pathway and modulating the expression of hippocampal synaptic plasticity-related proteins. Panaxynol, commonly found in plants of the *P. ginseng* C.A.Mey., can alleviate HPA axis overactivity induced by CUMS, promote the release of 5-HT and dopamine (DA), enhance hippocampal synaptic plasticity, and improve neurotransmitter effectiveness (Table 2 showed the active metabolites of TCM information) (Sun et al., 2020).

**TABLE 2:** Information on the action of active metabolites in TCM.

Category	Active metabolites	Source information	*In vivo*/*in vitro*	Modeling method	Dosage	Behavioral testing evaluation	Main pharmacological mechanisms	References
Flavonoids	Engeletin	*Astragalus mongholicus Bunge*	*In vivo*	CRS	2.5, 5, 10, 20 mg/kg	FST, TST, OFT, SPT	Activation of BDNF/TrkB/mTORC1 signaling pathway and regulation of PFC synaptic plasticity	Xu et al. (2023)
	Baicalein	*Scutellaria baicalensis* Georgi	*In vivo*	Rotenone	300 mg/kg	TST, SPT, OFT, Rotarod test	Activating the BDNF/TrkB/CREB signaling pathway to improve neural plasticity	Zhao et al. (2021)
			*In vivo* and *in vitro*	LPS	*In vivo*: 3 mg/kg	FST, TST	Increase the proportion of mBDNF/proBDNF to regulate neuronal survival and synaptic plasticity	Liu et al. (2022)
	Baicalin	*Scutellaria baicalensis* Georgi	*In vivo*	CMS	25, 50, 100 mg/kg	OFT, FST, SPT	Promote the expression of BDNF and CREB, regulate neuronal survival and synaptic plasticity	Lu et al. (2019)
	Quercitrin	Multiple fruits and vegetables	*In vivo*	LPS	10, 20, 30 mg/kg	FST, TST, OFT, SPT	Inhibiting Neuroinflammation PI3K/AKT/NF-κB Signal Transduction and Improving Damaged CREB/BDNF Neuroplastic Signal Transduction	Sun et al. (2021)
	luteolin	*Chrysanthemum indicum L.*, *Capsicum annuum L.*, and *Perilla frutescens (L.) Britton*	*In vivo*	CUMS	25 mg/kg	SPT, OFT, FST, MWM	Regulating Neuroplasticity Related Proteins	Liu et al. (2023)
	Soy isoflavones	*soybeans*	*In vivo*	LPS	10, 20, 40 mg/kg	FST, TST, OFT, SPT	Upregulation of hippocampal SYP phosphorylation and expression of PSD95	Lu et al. (2022)
Polyphenols	Curcumin	*Curcuma longa L*	*In vivo*	Ovariectomised	100 mg/kg	FST	Regulating neural plasticity	Abd-Rabo et al. (2019)
			*In vivo*	CUMS	40 mg/kg	OFT, SPT, FST	Regulating neural plasticity related proteins (PSD95 and SYP)	Zhang et al. (2014)
Alkaloids	Berberine	*Coptis chinensis* Franch	*In vivo*	CUMS	2.5, 5, 10 mg/kg	OFT, FST, Novelty-suppressed feeding test (NSFT)	Promote synaptic plasticity and regulate tryptophan metabolism by inhibiting IDO1 and activating TPH1	Ge et al. (2023)
			*In vivo*	CORT	100, 200 mg/kg	FST, TST, OFT, SPT	Inhibiting the activation of NLRP3 inflammasome to reduce neuroinflammatory response and promote synaptic plasticity and neurogenesis	Qin et al. (2023)
Saponins	Saikosaponin C	*Bupleurum chinense DC.*	*In vivo* and *in vitro*	*In vivo*: CSDS *In vitro*: LPS/ATP	*In vivo*: 0.5, 1 mg/kg	Social interaction TEST (SI), SPT, TST, FST, OFT	Inhibiting DNMT1 protein to reduce IL6 methylation, inducing decreased IL6 expression, and promoting synaptic plasticity	Bai et al. (2023)
	Ginsenoside Rb1	*Panax ginseng* C.A.Mey	*In vivo*	CUMS	20 mg/kg	FST, TST, OFT, SPT	Regulating hippocampal synaptic plasticity through the miR-134 mediated BDNF pathway	Wang et al. (2022b)
			*In vivo* and *in vitro*	*In vivo*: CUMS *In vitro*: LPS-ATP stimulation	*In vivo*: 10 mg/kg	OFT, FST, SPT	Regulating mitophagy and NF-κB pathway to inhibit astrocyte pyroptosis, thereby inhibiting neuroinflammation and enhancing synaptic plasticity	Li et al. (2023a)
	Ginsenoside-Rg1	*Panax ginseng* C.A.Mey	*In vivo*	LPS	40 mg/kg	SPT, FST, OFT, EPM, MWM	It has a synergistic effect with volumetric running, with anti-inflammatory and improved neural plasticity functions	Wang et al. (2023a)
Terpenoids	Geniposide	*Gardenia jasminoides* J.Ellis	*In vivo*	prenatal restraint stress	25, 50, 100 mg/kg	SPT, OFT, FST	Regulating the HPA axis and improving the expression of synaptic plasticity related proteins	Ma et al. (2024)
	Paeoniflorin	*Paeonia lactiflora Pall*	*In vivo*	CUMS	20 mg/kg	SPT, FST, TST, MWM	Improving LTP in hippocampal CA1 region and upregulating hippocampal dendritic spine density and expression levels of BDNF and PSD95	Liu et al. (2019)
	Oleanolic acid	*Olea europaea L*	*In vivo*	Maternal separation	30 mg/kg	OFT, EPM, Splash test, FST	Both OA and UA can upregulate synapsin levels, and OA can also upregulate the expression level of PSD95	Kong et al. (2023)
	Ursolic acid	Exists in various plants						
Polysaccharides	Polysaccharides from *Polygonatum cyrtonema* Hua	*Polygonatum cyrtonema Hua*	*In vivo*	Single prolonged stress	200, 400, 800 mg/kg	OFT, EPM, Fear conditioning task	Relieve oxidative stress and neuroinflammation, and act on the Nrf2/HO-1 signaling pathway to improve synaptic damage	Xie et al. (2024)
	Inulin	*Inula helenium L*	*In vivo*	CUMS	0.037 g of inulin/kcal	SPT, FST, OFT, TST, EPM	Enhancing CREB/BDNF signaling to improve neurogenesis and synaptic plasticity	Wang et al. (2023b)
	Polysaccharide-rich fraction from *Schisandra chinensis* (Turcz.) Baill	*Schisandra chinensis* (Turcz.) Baill	*In vivo*	Olfactory bulbectomy	50, 200, 800 mg/kg	FST, TST, Locomotor activity test	Improving abnormal synaptic plasticity (upregulating PSD95 expression), inhibiting excessive HPA axis activity, and regulating gut microbiota	Zhu et al. (2024)
Botanical drugs extracts	Saffron Extract (Affron^®^)	Saffron (*Crocus sativus L.*)	*In vivo*	Unpredictable chronic mild stress	100, 200 mg/kg	SPT	Adjusting the HPA axis to increase hypothalamic neural plasticity	Kim et al. (2023)
	Blueberry Extract	Blueberry (*Vaccinium* spp.)	*In vivo*	LPS	100, 200 mg/kg	OFT, FST	Downregulation of hippocampal AChE activity, inhibition of neuroinflammation, and potential protection of neuroplasticity	Spohr et al. (2023)
Other types	Honokiol	*Magnolia grandiflora L*	*In vivo* and *in vitro*	*In vivo*: CUMS	10 mg/kg	OFT, SPT	Activate the HIF-1α/VEGF signaling pathway and upregulate the protein expression levels of SYP 1 and PSD 95	Fan et al. (2022)
	Salidroside	*Rhodiola rosea L*	*In vivo* and *in vitro*	*In vivo*: CORT or LPS *In vitro*: CORT or nigericin	20, 40 mg/kg	OFT, SPT, FST	Upregulation of BDNF expression, improvement of synaptic plasticity, and inhibition of P2X7/NF-κB/NLRP3 signaling pathway mediated pyroptosis	Chai et al. (2022)
	Crocin	*Crocus sativus L*	*In vivo* and *in vitro*	*In vivo*: CUMS	12.5, 25 mg/kg	SPT, TST, FST, OFT	Regulating the Wnt/β-catenin signaling pathway to promote adult hippocampal neurogenesis	Tao et al. (2023a)
			*In vivo*	Prenatal stress	10, 20, 40 mg/kg	OFT, TST, FST, SPT, NSFT	Regulating hippocampal synaptic plasticity related proteins	Wu et al. (2020)
	Panaxynol	*Panax ginseng* C.A.Mey	*In vivo*	CUMS	1.0 mg/kg	OFT, EPM, SPT	Regulating the HPA axis, promoting the release of 5-HT and DA, and improving hippocampal synaptic plasticity	Sun et al. (2020)

### 5.2 TCM formulas

Zhi-Zi-Chi-Tang (ZZCT) is a potent traditional Chinese herbal remedy with a historical record in the “Shanghan Lun.” It consists of the dehydrated mature fruits of *G. jasminoides* J. Ellis and *G. max* (L.) Merr. In a rat depression model induced by CUMS, ZZCT enhances neuroplasticity through the 14–3–3ζ/GSK-3β/CREB/BDNF signaling pathway. It restores the expression of synaptic plasticity-related proteins like MAP2 and PSD95 in the hippocampal CA1 region, enhances LTP induction, and improves neuronal damage caused by CUMS (Tao et al., 2023b).

Zi-Shui-Qing-Gan-Yin (ZSQGY) is another traditional Chinese herbal remedy commonly used in China for depression symptoms. ZSQGY consists of 12 botanical drugs, including *P. ginseng* C.A.Mey et al. In a study conducted both *in vivo* and *in vitro* by Zhu et al. (2023), it was found that ZSQGY significantly improved depression-like behavior induced by monosodium glutamate (MSG) in rats. Further investigations revealed that ZSQGY improved synaptic ultrastructure by upregulating PGC-1α, regulating mitochondrial function, and inhibiting the expression of pro-inflammatory cytokines (Zhu et al., 2023).

The traditional remedy Danggui-Buxue Decoction (DBD), is taken from Li Dongyuan’s work on differentiating endogenous and exogenous diseases in the Jin and Yuan Dynasties (Shi et al., 2019). Studies suggest that DBD protects and reshapes hippocampal neurons by regulating the CREB/BDNF/TrkB pathway. It shows promise as a potential metabolite for preventing diabetes mellitus with depression (DD), with ferric acid potentially playing a crucial role in its effects (Wang et al., 2021). The Erzhi formula, composed of *Ligustrum lucidum* W.T.Aiton and *Eclipta prostrata* (L.) L., represents a TCM treatment (Peng et al., 2022). In an *in vitro* depression model, the Erzhi formula revealed the capacity to diminish dexamethasone-induced apoptosis in primary cultured cortical neurons and repair synaptic damage. Its neuroprotective effects were linked to the 11β-hydroxysteroid dehydrogenase 1 (HSD1)-glucocorticoids (GC)/glucocorticoid receptor (GR) signaling pathway (Han et al., 2023).

Xiaoyaosan, a TCM formula first introduced in the book “Prescriptions of the Bureau of Taiping People’s Welfare Pharmacy,” has a historical use in treating mental disorders, such as depression (Jiao et al., 2024). The ancient Chinese medicine pharmacopoeia also mentions Jiawei-Xiaoyao pill (JWX), a traditional Chinese medication, for the treatment of a variety of illnesses, including mood disorders. JWX consists of nine botanical drugs, including *G. jasminoides* J. Ellis et al. Studies have shown that JWX stimulates CaMKII signaling, leading to the activation of the mTOR/BDNF signaling pathway, Furthermore, it also enhances hippocampal neuroplasticity and triggering rapid antidepressant effects (Zhang et al., 2024a).

For more precise administration in patients with depression, Gao et al. (2018) introduced an empirical prescription called modified Xiaoshan (MXYS) based on Xiaoshan consisting of *B. chinense* DC et al. In a depression model induced by CUMS, MXYS was found to promote hippocampal neurogenesis and improve brain blood oxygen level-dependent signaling, indicating its potential therapeutic benefits for depression (Gao et al., 2018).

SiNiSan (SNS) is a TCM formula. Originally mentioned in the Treatise on Febrile Diseases for controlling liver qi (Cao et al., 2024), SNS has been shown to regulate neuroplasticity by activating the Calcium sensitive receptor (CaSR)-protein kinase C (PKC)-ERK signaling pathway. It also helps in regulating mitochondrial function and improving neuroplasticity (Shen et al., 2020b; Deng et al., 2022). Suanzaoren Decoction (SZRD), a TCM combination with a history of insomnia treatment (Dong et al., 2021; Yan et al., 2023b). Research by Du et al. (2024) using *in vivo* and *in vitro* experiments demonstrated that SZRD increases the expression levels of BDNF, SYP, and PSD95. It also inhibits the activation of the TLR4/MyD88/NF-κB and Wnt/β-catenin pathways, showing antidepressant effects, and SZRD could also adjust the CaMK signal system (Zhang et al., 2024b; Du et al., 2024).

**FIGURE 3:**
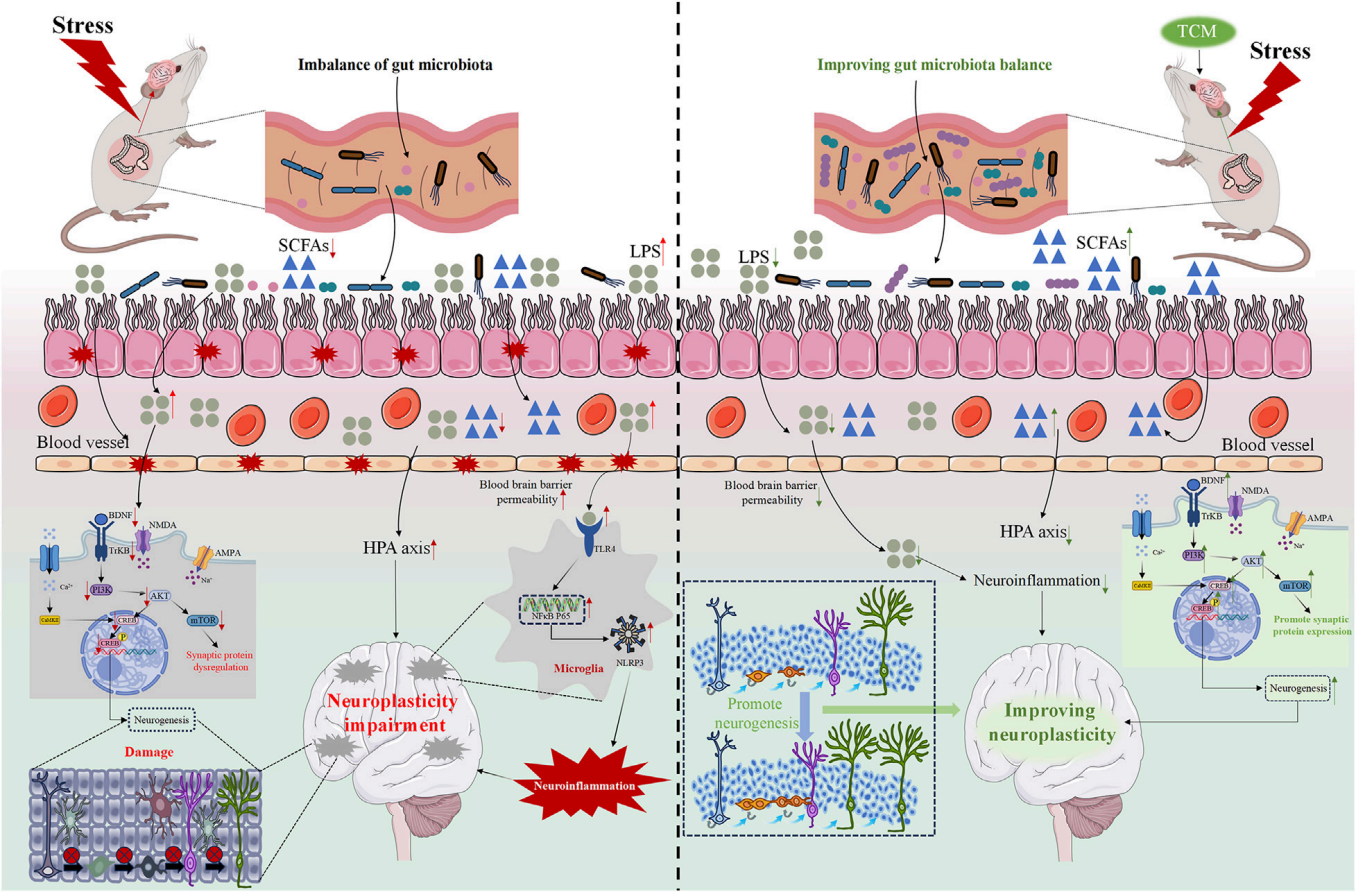
The pharmacological mechanism of TCM regulation of neuroplasticity in the treatment of depression. The red arrow indicates changes caused by stress, while the green arrow indicates changes caused by TCM.

Zhi-Zi Hou-Po Decoction (ZZHP), a TCM formula widely used in depression treatment (Feng et al., 2022). Studies suggest that ZZHP effectively reverses the decrease of monoamine neurotransmitters in the hippocampus, maintains their homeostasis, activates the BDNF/TrkB/CREB pathway, protects neuronal synaptic plasticity, promotes hippocampal neurogenesis, and alleviates depression-like symptoms in mice caused by CUMS (Ye et al., 2024). Kaiyu Zhishen Decoction (KZD) is composed of botanical drugs such as *P. lactiflora* Pall. Chen et al. (2024) found through network pharmacology and experimental verification that the antidepressant effect of KZD involves regulating the ERK-CREB-BDNF signaling pathway and promoting neuronal repair, potentially regulating neuroplasticity (Figure 3 showed the mechanism of TCM action and Table 3 showed the TCM formulas information).

**TABLE 3:** Information on the action of TCM formulas.

TCM formulas	Main composition	*In vivo*/*in vitro*	Modeling method	Dosage	Behavioral testing evaluation	Main pharmacological mechanisms	References
Zhi-Zi-Chi-Tang (ZZCT)	*Gardenia jasminoides* J.Ellis and *Glycine max (L.)* Merr	*In vivo*	CUMS	3, 6 g/kg	SPT, TST, FST, OFT	Regulating the 14–3–3ζ/GSK-3β/CREB/BDNF signaling pathway to enhance neural plasticity	Tao et al. (2023b)
Zi-Shui-Qing-Gan-Yin (ZSQGY)	*Panax ginseng C.A.Mey*, *Dioscorea oppositifolia L*, *Bupleurum chinense DC*, *Paeonia lactiflora Pall*, *Angelica sinensis (Oliv.) Diels*, *Anemarrhena asphodeloides Bunge*, Cornus officinalis Siebold and Zucc, Paeonia × suffruticosa Andrews, *Smilax glabra Roxb, Ziziphus jujuba Mill*, *Alisma plantago-aquatica L* and *Gardenia jasminoides* J.Ellis	*In vivo* and *in vitro*	*In vivo*: monosodium glutamate *In vitro*: CORT	12, 24, 48 g/kg	SFT, SPT, OFT	Upregulation of PGC-1α to improve pathological changes in synaptic ultrastructure, regulate mitochondrial function, and inhibit the expression level of pro-inflammatory cytokines	Zhu et al. (2023)
Danggui-Buxue Decoction (DBD)	*Astragalus mongholicus Bunge* and *Angelica sinensis (Oliv.) Diels*	*In vivo*	CUMS	4, 8 g/kg	FST, OFT, TST	Regulating the CREB/BDNF/TrkB pathway to protect and reshape hippocampal neurons	Wang et al. (2021)
Erzhi formula	*Ligustrum lucidum W.T.Aiton* and *Eclipta prostrata (L.) L*	*In vitro*	Dexamethasone	--	--	Reduce neuronal apoptosis and improve synaptic damage	Han et al. (2023)
Jiawei-Xiaoyao pill (JWX)	*Gardenia jasminoides J.Ellis*, *Paeonia × suffruticosa Andrews*, *Bupleurum chinense DC.* , *Paeonia lactiflora Pall.*, *Angelica sinensis (Oliv.) Diels*, *Atractylodes macrocephala Koidz.*, *Smilax glabra Roxb.*, *Glycyrrhiza glabra L.* and *Mentha canadensis L*	*In vivo*	CORT	0.7, 1, 1.4, 1.8 g/kg	OFT, TST, FST, SPT	Stimulation of CaMKII signaling pathway, followed by activation of mTOR/BDNF signaling pathway, enhances hippocampal neural plasticity	Zhang et al. (2024a)
Modified Xiaoyaosan (MXYS)	*Bupleurum chinense DC.*, *Angelica sinensis (Oliv.) Diels*, *Paeonia lactiflora Pall.*, *Atractylodes macrocephala Koidz.*, *Acorus calamus L.*, *Curcuma aromatica Salisb.*,*Reynoutria multiflora (Thunb.)* Moldenke, *Schisandra chinensis (Turcz.) Baill.*, *Ziziphus jujuba Mill.*, and *Periploca forrestii Schltr*	*In vivo*	CUMS	0.4 g/kg	SPT, TST, FST	Promote hippocampal neurogenesis and improve BOLD signaling	Gao et al. (2018)
SiNiSan (SNS)	*Citrus × aurantium f. Aurantium*, *Paeonia lactiftora Pall.*, *Glycyrrhiza glabra L.*, and *Bupleurum chinense DC*	*In vivo*	Maternal separation and CUMS	0.25, 0.5, 1 g/mL	SPT, OFT, FST	Activating the CaSR-PKC-ERK signaling pathway	Shen et al. (2020b)
		*In vivo*	Maternal separation	2.5, 5, 10 g/kg	SPT, OFT, FST	Regulating mitochondrial function, and improving neural plasticity	Deng et al. (2022)
Suanzaoren Decoction (SZRD)	*Ziziphus jujuba Mill.*, *Smilax glabra Roxb.*, *Anemarrhena asphodeloides Bunge*, *Oreocome striata (DC.) Pimenov and Kljuykov*, and *Glycyrrhiza glabra L*	*In vivo* and *in vitro*	*In vivo*: CUMS *In vitro*: LPS	15 g/kg	SPT, FST, OFT	Elevated the expression levels of BDNF, SYP, and PSD95, and inhibited the activation of TLR4/MyD88/NF-κB and Wnt/β-catenin pathways	Du et al. (2024)
		*In vivo*	CUMS	2.5, 5, 10 g/kg	SPT, OFT	Modulating CaMK signal system	Zhang et al. (2024b)
Zhi-Zi Hou-Po Decoction (ZZHP)	*Gardenia jasminoides J.Ellis*, *Citrus × aurantium f. Aurantium* and *Magnolia officinalis Rehder and E.H.Wilson*	*In vivo*	CUMS	0, 30, 40 mg/kg	SPT, TST, FST, OFT	Activating the BDNF/TrkB/CREB pathway protects neuronal synaptic plasticity and promotes hippocampal neurogenesis	Ye et al. (2024)
Kaiyu Zhishen Decoction (KZD)	*Paeonia lactiflora Pall., Cyperus rotundus L., Smilax glabra Roxb., Angelica sinensis (Oliv.) Diels., Panax ginseng C.A.Mey., Gardenia jasminoides J.Ellis., Atractylodes macrocephala Koidz., Citrus reticulata Blanco., Glycyrrhiza glabra L.,* and *Bupleurum chinense DC*	*In vivo* and *in vitro*	*In vivo*: CUMS *In vitro*: CORT	1.579, 4.73, 14.21 g/kg	SPT, FST, TST	Regulating the ERK-CREB-BDNF signaling pathway and enhancing neuronal repair	Chen et al. (2024)

## 6 Conclusion and prospects

Depression is a common long-lasting mental disorder marked by enduring feelings of sadness, low self-esteem, and potentially dangerous suicidal ideation. Understanding the pathogenesis of depression remains a challenge in modern medicine, and there is a deficiency of therapeutic strategies that may effectively prevent or entirely reverse depression (Chen et al., 2022b; Xia et al., 2023). At now, great progress has been achieved in the study of depression, both at the preclinical level and at the fundamental research level. Multiple chemicals with antidepressant effects have been developed in some clinical treatments, but there are still certain side effects and insufficient efficacy. In addition, there is a lack of suitable and appropriate depression prediction tools in clinical practice. Currently, finding antidepressant drugs with multiple targets, high safety, good efficacy, and minimal adverse reactions is a major task.

In recent years, TCM has received attention and promotion, and has been vigorously developed in various aspects. In the research of antidepressants, TCM has gradually become the focus and hotspot of research. In the treatment of depression, it is crucial to explore how TCM can complement Western medicine approaches, leveraging the strengths of TCM’s multi-target effects and individualized treatments. Research in multi-target antidepressant therapies is essential to achieve outcomes comparable to modern medical “cocktail therapy.” TCM offers multiple advantages, including its emphasis on multiple targets and individualized treatment in line with the principles of precision medicine. Active metabolites in TCM, such as flavonoids, polyphenols, alkaloids, saponins, terpenes, polysaccharides, and TCM extracts, along with TCM formulas such as ZZCT, ZSQGY, DBD, Erzhi formula, JWX, MXYS, SNS, SZRD, KZD and ZZHP, play a role in regulating neuroplasticity through various targets and pathways when exerting antidepressant effects.

However, the causes and mechanisms of depression have not been fully elucidated, and there is a lack of unified and relatively authoritative methods for evaluating depression symptoms in clinical practice. There is no clear standard for the specific indicators of depression. More importantly, current research mostly focuses on the *in vivo* or *in vitro* levels, lacking high-quality clinical research on active metabolites and TCM formulas. Most studies only explore the mechanism of drug action, and the connection between TCM theory and neuroplasticity has not been thoroughly investigated. Furthermore, compared to the active metabolites of TCM, research on TCM formulas is relatively weak, and the diversity and depth of neuroplasticity-related signaling pathways explored are insufficient. There is no active substance in the world that not only exerts its pharmacological effects but also has non-specific off-target effects on normal tissues of the body (Guo et al., 2023). In current research on antidepressants, there has been insufficient exploration of the toxicology and side effects of TCM.

In addition, some Chinese herbal medicines lack clear quality control standards, compromising the stability and consistency of their chemical metabolites, limiting their clinical application and complicating the study of their pharmacological mechanisms. Furthermore, certain active metabolites of TCM face challenges such as poor stability, solubility issues, and difficulty in crossing the blood-brain barrier, which need further investigation to ascertain their efficacy in targeting CNS organs. The mechanism of neuroplasticity is complex, involving multiple signaling pathways and cell coordination. While TCM possesses the advantage of targeting multiple pathways, current research predominantly focuses on single signaling pathways with limited detection indicators. This approach fails to comprehensively elucidate the synergistic mechanisms underlying TCM’s multi-target and multi-pathway regulation of neuroplasticity.

Therefore, in future research, multicenter, large-sample clinical randomized controlled trials guided by TCM theory should be conducted to explore the efficacy and safety of TCM in treating depression, as well as the regulatory mechanisms of neuroplasticity, aiming to provide deeper insights into how TCM works in antidepressant treatment. Simultaneously, it is essential to enhance the quality control standards for TCM and strengthen the exploration of targeted delivery systems for TCM to increase the concentration and duration of TCM in target organs, thereby improving the therapeutic outcomes. Furthermore, focusing on cutting-edge technologies such as combined single-cell sequencing and spatial transcriptomics is necessary to further reveal the key regulatory targets of TCM and the regulatory mechanisms of neuroplasticity at different time points and cell types. This review systematically elucidated the role of neuroplasticity in the pathological development of depression and the regulatory role of TCM. In conclusion, substantial research efforts are still needed to fully explore the potential of TCM in modulating neuroplasticity for the prevention and treatment of depression.

## Figures and Tables

**Table:** 

**TCM**	Traditional Chinese medicine
**MDD**	Major depressive disorder
**SSRIs**	Selective serotonin reuptake inhibitors
**LTP**	Long-term potentiation
**LTD**	long-term depression
**BDNF**	Brain-derived neurotrophic factor
**TrkB**	Tyrosine kinase receptor B
**cAMP**	Cyclic adenosine monophosphate
**CREB**	cAMP responsive element binding protein
**CUMS**	Chronic unpredictable mild stress
**MS**	maternal-infant separation
**DG**	Dentate gyrus
**CSIS**	Chronic social isolation
**HPA**	Hypothalamic-pituitary-adrenal
**ACTH**	Adrenocorticotropin
**CRH**	Corticotropin-releasing hormone
**CORT**	Cortisol
**BNIP3L**	BNIP3 like
**LPS**	Lipopolysaccharide
**PNs**	Projection neurons
**CNS**	Central nervous system
**BLA**	Basolateral amygdala
**nNOS**	neural nitric oxide synthase
**GAP-43**	Growth associated protein 43
**5-HT**	Serotonin
**NMDAR**	N-methyl-D-aspartate receptor
**4E-BPs**	4E binding proteins
**AR**	Allergic rhinitis
**H**_**2**_ **S**	Hydrogen sulfide
**SI**	Soy isoflavones
**p-SYP**	Phosphorylated SYP
**OA**	Oleanolic acid
**PTSD**	Post-traumatic stress disorder
**SCFAs**	Short-chain fatty acids
**AChE**	Acetylcholinesterase
**PNS**	Prenatal stress
**DA**	Dopamine
**ZZCT**	Zhi-Zi-Chi-Tang
**ZSQGY**	Zi-Shui-Qing-Gan-Yin
**MSG**	monosodium glutamate
**DBD**	Danggui-Buxue Decoction
**AR**	*Astragali Radix*
**ASR**	*Angelica Sinensis Radix*
**DD**	Diabetes mellitus with depression
**HSD1**	11β-hydroxysteroid dehydrogenase 1
**GC**	Glucocorticoids
**GR**	Glucocorticoid receptor
**JWX**	Jiawei-Xiaoyao pill
**MXYS**	Modified Xiaoshan
**SNS**	SiNiSan
**SZRD**	Suanzaoren Decoction
**ZZHP**	Zhi-Zi Hou-Po Decoction
